# COVID-19 infections in staff of an emergency care hospital after the first wave of the pandemic in Germany

**DOI:** 10.3205/dgkh000407

**Published:** 2022-03-01

**Authors:** Philipp Stüven, Georg Mühlenbruch, Agnes Evenschor-Ascheid, Ellen Conzen, Claudia Peters, Anja Schablon, Albert Nienhaus

**Affiliations:** 1 University Hospital Hamburg-Eppendorf (UKE), Institute for Health Services Research in Dermatology and Nursing (IVDP), Centre for Epidemiology and Health Services Research for Healthcare Professionals (CVcare), Hamburg, Germany; 2 Rhine-Maas Hospital, District Aachen, Würselen, Germany; 3 German Statuary Institution for Accident Insurance and Prevention for Health and Welfare Services (BGW), Department of Occupational Medicine, Toxic Substances, Health Service Research, Hamburg, Germany

**Keywords:** occupational health, COVID-19, infection risk, health worker, hospital

## Abstract

**Background:** Hospital staff have an increased risk of SARS-CoV-2 infection. It is thus necessary to monitor the situation because infected staff may in turn infect patients and their family members. Following the first wave of infection in the summer of 2020, the Rhine-Maas Hospital (RMK) provided all staff the opportunity to be tested for SARS-COV-2 via antibody testing.

**Methods:** The tests were carried out from 19.6.2020 to 17.7.2020. The IgG antibody test qualitatively tested for SARS-CoV-2 antibodies via enzyme-linked immunosorbent assay (ELISA). An IgG titre of 0.8 IU/mL or more was considered positive. All staff who tested positive for SARS-CoV-2 by PCR testing after February 2020 were also included in the study. Occupational and non-occupational risk factors for infection were determined. Staff in the intensive care ward, the emergency depart-ment, or a SARS-CoV-2 ward (“corona ward”) were predefined as having increased exposure. Odds ratios (OR) were calculated using logistical regression for occupational and private infection risk.

**Results:** 903 staff members (58.9%) with complete data took part in the cross-sectional study. 52 staff members (5.8%) had a positive PCR test result in their medical history or tested positive in the IgG test. Around half of the infections (55%) were only detected by serological testing during the study. Staff with tasks classified as at-risk had an OR of 1.9 (95% CI 1.04–3.5) for infection. Risk factors also included private contacts to people infected with SARS-CoV-2 and holidays in risk areas. At the time of data collection, 11.5% of those with the disease reported that they had not yet fully recovered from COVID-19.

**Discussion:** Following the first COVID-19 wave, 5.3% of staff at the RMK were infected. An increase in occupational infection risk was found even after controlling for non-occupational infection risks. This should be taken into account with regard to the recognition of COVID-19 as an occupational disease. Methods to improve protection against nosocomial transmissions should be considered.

## Zusammenfassung

**Hintergrund:** Beschäftigte im Krankenhaus haben ein erhöhtes Risiko für eine SARS-CoV-2-Infektion. Ein Monitoring der Situation ist auch deshalb notwendig, weil infizierte Beschäftigte sowohl Patienten als auch ihre Angehörigen infizieren können. Am Rhein-Maas-Klinikum (RMK) wurde deshalb nach der ersten Infektionswelle im Sommer 2020 allen Beschäftigten ein Angebot gemacht, sich auf SARS-CoV-2 mittels Antikörpertest untersuchen zu lassen.

**Methoden:** Die Untersuchungen wurden vom 19.6.2020 bis zum 17.7.2020 durchgeführt. Der IgG-Antikörpertest erfolgte mit Enzyme-linked Immunosorbent Assay (ELISA) qualitativ auf SARS-CoV-2-Antikörper. Ein IgG-Titer ab 0,8 IU/ml wurde als positiv bewertet. Ferner wurden alle Beschäftigten, die seit Februar 2020 mittels PCR auf SARS-CoV-2 positiv getestet worden waren, in die Studie eingeschlossen. Erhoben wurden berufliche und außerberufliche Risikofaktoren für eine Infektion. Beschäftigte auf der Intensivstation, in der zentralen Notaufnahme oder auf einer SARS-CoV-2-Station („Corona-Station“) wurden vorab als erhöht Exponierte eingestuft. Für das berufliche und private Infektionsrisiko wurden Odds Ratios (OR) in einer logistischen Regression berechnet.

**Ergebnisse:** An der Querschnittsstudie nahmen 903 Beschäftigte (58,9%) mit vollständigen Daten teil. Eine positive PCR in der Anamnese oder einen positiven IgG-Test wiesen 52 Beschäftigte (5,8%) auf. Etwa die Hälfte der Infektionen (55%) wurde erst im Rahmen der Studie mit der Serologie entdeckt. Beschäftigte mit Tätigkeiten, die als gefährdend eingestuft wurden, hatten ein OR von 1,9 (95%-CI 1,04–3,5) für eine Infektion. Private Kontakte zu SARS-CoV-2-Infizierten und Urlaube in Risikogebieten waren ebenfalls Risikofaktoren. Zum Zeitpunkt der Erhebung gaben 11,5% der Erkrankten an, sich noch nicht wieder vollends von COVID-19 erholt zu haben.

**Diskussion:** Nach der ersten COVID-19-Welle waren 5,3% der Beschäftigten des RMK infiziert. Auch nach der Kontrolle für außerberufliche Infektionsrisiken war das beruflich bedingte Infektionsrisiko erhöht. Das sollte bei der Anerkennung von COVID-19 als Berufskrankheit berücksichtigt werden. Möglichkeiten zur Verbesserung des Schutzes vor nosokomialen Übertragungen sollten erwogen werden.

## Key points

- After the first wave of the COVID-19 pandemic, prevalence of IgG indicating SARS-CoV-2 infection was low in hospital workers in Germany.
- About half of the infections discovered by serology were not detected before by symptoms and PCR test.
- Working in the emergency room, intensive care or at the COVID-19 ward was a risk factor for infection, even after controlling for private risk factors.
- Symptoms of Long COVID were indicated by about 10% of the infected health workers.

## Background

Cases of illness with a novel coronavirus (SARS-CoV-2) were identified for the first time in China in December 2019. Within a few months, the virus had triggered a worldwide pandemic that is still ongoing. The typical symptoms of a SARS-CoV-2 infection are cough, rhinitis, sore throat and fever. The loss of smell and taste have also been frequently reported. Severe symptoms may lead to fatal complications caused by acute interstitial pneumonia. The first sign of this is usually having difficulty breathing. The disease with its wide range of symptoms is classified as COVID-19. The first case in Germany was identified on 27 January 2020. Over 1.7 million people contracted the disease in Germany that year, of whom more than 33,000 died in connection with a SARS-CoV-2 infection [1]. To this day, the disease continues to push the health system to its limits with unforeseeable conse-quences. People who work in jobs on the front line against the virus, such as doctors and nurses, seem to be at significantly greater risk of contracting COVID-19 themselves. As of May 2020, 152,888 infections and 1,413 deaths of healthcare workers (HW) were reported worldwide due to COVID-19 [2]. Several studies have shown an increased prevalence in healthcare workers compared to the general public [3], [4]. In a European study, infection risk for HW was compared to the infection risk of the general population. The adjusted attack rate ratio in HWs (compared with non-HWs) was 3.0 (95% confidence interval (CI) 2.2–4.0) for infection [5]. Sick leave or hospitalisation due to COVID-19 was 2.4 times more frequent in these occupations than in all other occupations, according to data from German health insurers [6]. Therefore, every effort should be made to stop the spread of the disease among HW and to address any potential risk factors. This can be achieved by following general hygiene rules, wearing additional personal protective equipment (PPE), and vaccinating against COVID-19. At the same time, possible mutations that require further measures, e.g., adaptation of the vaccines, should also be kept in mind.

In order to assess the situation for hospital staff, we conducted a cross-sectional study at the Rhine-Maas hospital (RMK) in the Aachen district after the first wave in summer 2020. The RMK is a primary care hospital. It is located in the vicinity of the Heinsberg district, which was particularly affected by the first wave of infection. This meant that investigating the course of infection at the beginning of the pandemic among RMK staff was of particular interest. We intended to analyse the prevalence of SARS-CoV-2 infections in HW of the hospital and to analyse occupational and private risk factors for infection. 

## Methods

### Study design and participants

The cross-sectional study was conducted from 19.6.2020 to 17.7.2020. At the time of data collection, the hospital had 1,532 staff members and 663 hospital beds (total capacity including geriatric rehabilitation). This study was preceded by an outbreak event in February 2020. At the time, staff with typical COVID-19 symptoms or with known contacts to infected patients were tested by PCR, but a systematic investigation of all staff was not carried out. In the cross-sectional study, no PCR was performed, but rather specific antibodies were determined. PCR testing was not carried out because the incidence of SARS-CoV-2 infection in the population was low at the time of the study, and the logistical burden of PCR testing for all staff did not seem justified. 

The cross-sectional study was conducted as a prevalence study in the context of an occupational health check-up. All RMK hospital staff were informed of the study’s objective on several occasions via email and intranet. Participation was voluntary. All staff were given the opportunity to take part in the study irrespective of whether they worked closely with patients. There were no exclusion criteria. No financial or other incentives were given for participating in the study. In-person briefings were held in addition to the participant information provided on the intranet. All participants gave their written consent in advance. The German Statutory Institution for Accident Insurance and Prevention for Health and Welfare Services (BGW) provided funding. The Ethics Committee of the Medical Association of Hamburg (Ethics Committee Application No. PV7298) approved the study. 

Blood samples (1 serum monovette) were taken from all participants for the study. The RMK provided staff for the coordination and collection of the blood samples, as well as the collection and encryption of data. The occupational health doctor (A. E-A) and her staff carried out the blood collection specifically. In addition, staff working in various departments also performed blood collection. A phlebotomy team (staff responsible only for blood collection at the RMK) also supported the blood collection. 

The serology evaluation was performed by an external laboratory that has cooperated with the hospital for years and has a branch at the RMK. Some of the laboratory staff and the laboratory manager are also staff of the RMK. 

Blood samples were qualitatively tested for SARS-CoV-2 antibodies (IgG and IgA) using enzyme-linked immunosorbent assay (ELISA), in accordance with the manufacturer’s instructions [7]. IgG values below 0.8 in the serological testing were rated as negative, and IgG values ≥0.8 were rated as positive. Participants with isolated detection of IgA were not considered positive due to insufficient specificity [7], [8].

All staff with a positive PCR result in their medical history and/or a positive IgG test result (≥0.8 IU/mL) were considered infected. Participants with IgG results in the range of 0.8 to <1.5 IU/mL and in the range of ≥1.5 were compared with respect to typical COVID-19 results [8], [9].

A standardized in-house questionnaire collected socio-demographic data as well as the risk of infection that the employees were exposed to, both at work and in their private lives. The questionnaire also included questions regarding visits to risk areas. 

All participants were divided into risk groups in order to better assess their risk of infection with SARS-CoV-2. Staff in the intensive care ward, the emergency department or a SARS-CoV-2 ward (“corona ward”) were pooled together as the group with a high risk of infection. All other staff working closely with patients were identified as being at medium risk of infection. A low risk of infection was assigned to working in administration, in the kitchen or in the IT and technical areas, which have little or no patient contact. Separation into the risk groups was carried out before blood collection, i.e., before the infection status was known.

### Statistical analysis

The data were evaluated using descriptive analysis. Positive SARS-CoV-2 cases, by PCR or IgG antibody testing, were compared to those who tested negative. Categorical variables were represented by absolute and relative frequencies; metric variables were represented by mean (MW), medin, standard deviation (SD) and range. Group differences were calculated with the chi-squared test, with Fisher’s exact test in the case of a low ell frequency or with a t-test in the case of metric variables. A trend test was performed for ordinal variables. Multiple logistic regression was performed for the outcome of SARS-CoV-2 infection (yes/no) and the level of estimated risk of infecion. We also considered age and gender as additional independent variables, as well as private contacts with people with SARS-CoV-2 infections and holidays in risk areas. The modelling was completed step by step. Only variables with a p-value ≥0.1 were included in the model. A p-value of ≥0.05 was deemed statistically significant. The statistical analysis was performed using SPSS (version 27).

## Results

### Participants and response rate 

A total of 925 staff members (response rate of 60.4%) of the RMK took part in the study. Eight participants were excluded due to failure to complete the questionnaires, and 14 participants were excluded due to missing information. Therefore, 903 staff members were included in the evaluation. The age of the participants ranged from 17 to 83 years (median 44 years, mean 43.5 years) (Table 1 (Tab. 1)). The advanced age of some study participants (n=4) is because volunteers were included. Once the risk of SARS-CoV-2 for people of advanced age became known, they were sent home immediately, both for their own protection and for the protection of the vulnerable patient groups. 74% of the staff consisted of women. The largest occupational group comprised nurses with 42.3% of participants, followed by doctors with 19.4%. The most common working areas were in non-surgical wards (19.7%), followed by intensive care wards (14.6%) and surgical wards (14.3%) (Table 1 (Tab. 1)). 

A total of 52 (5.8%) staff members tested positive for SARS-CoV-2. Prior to the cross-sectional study, 23 staff members were tested positive via PCR. Among them, 20 staff members (87%) had a positive IgG test result, and three had a negative IgG test result (13.0%). A further 29 staff members (3.2% of all participants), who were not initially diagnosed with SARS-CoV-2 infection by PCR, had a positive serology test result (IgG ≥0.8 IU/mL) (Table 2 (Tab. 2)).

### Risk factors for infection

The average age of a SARS-CoV-2 infected individual was 38.5 years. In contrast, the age of negatively tested individuals was 43.9 years (p=0.002) (Table 1 (Tab. 1)). Gender had no influence on the probability of a positive test result (men 7.7%, women 5.1%, p=0.146). Nursing assistants had the highest rate of positive results (10.3%), and those in cleaning, kitchen and other occupations had the lowest (1.6%). However, these differences were not statistically significant. The probability of infection was highest for emergency department staff (15.4%). This difference was statistically significant. The risk of infection was not elevated for staff on “corona wards” (6.9% versus 5.1%). There was a statistically significant trend for the number as well as the duration of contacts. Wearing personal protective equipment had no influence on the prevalence of SARS-CoV-2 infection (Table 1 (Tab. 1)).

The prevalence of SARS-CoV-2 among staff with an assessed low risk of infection did not differ from the prevalence among staff with a medium risk of infection (3.7% versus 3.9%) (not shown in table). Therefore, these two groups were combined. A high infection risk was found for 410 staff members (Table 3 (Tab. 3)). These consisted of nurses, doctors and cleaning staff (not shown in table). Staff with a high infection risk had nearly twice the infection risk compared to staff with an estimated low or medium infection risk (8.0% versus 3.9%). The OR adjusted for private contacts and holidays in risk areas is 1.9 (95% CI 1.3–4.2). After further controlling for age, the OR does not change, only the 95% CI changes (1.04–3.5), but remains statistically significant. Staff with multiple job-related contacts with COVID-19 patients or with contacts lasting longer than 15 minutes had an increased OR for infection (OR=2.2). However, the OR was no longer statistically significant after controlling for age. 

Persons with private contacts to SARS-CoV-2 infected persons had a statistically significantly higher risk of infection compared to those without private SARS-CoV-2 contacts (12.6% versus 4.6%, OR 2.8; 95% CI 1.6–5.7). Visiting a risk area was also associated with a SARS-CoV-2 infection (9.0% versus 5.0%). However, the adjusted OR was not statistically significant (OR 1.5; 95% CI 0.9–3.0) (Table 2 (Tab. 2)). However, when analysed dichotomously, the chi-squared test yielded a statistically significant p of 0.039.

### Staff with a positive test

**Figure 1:**
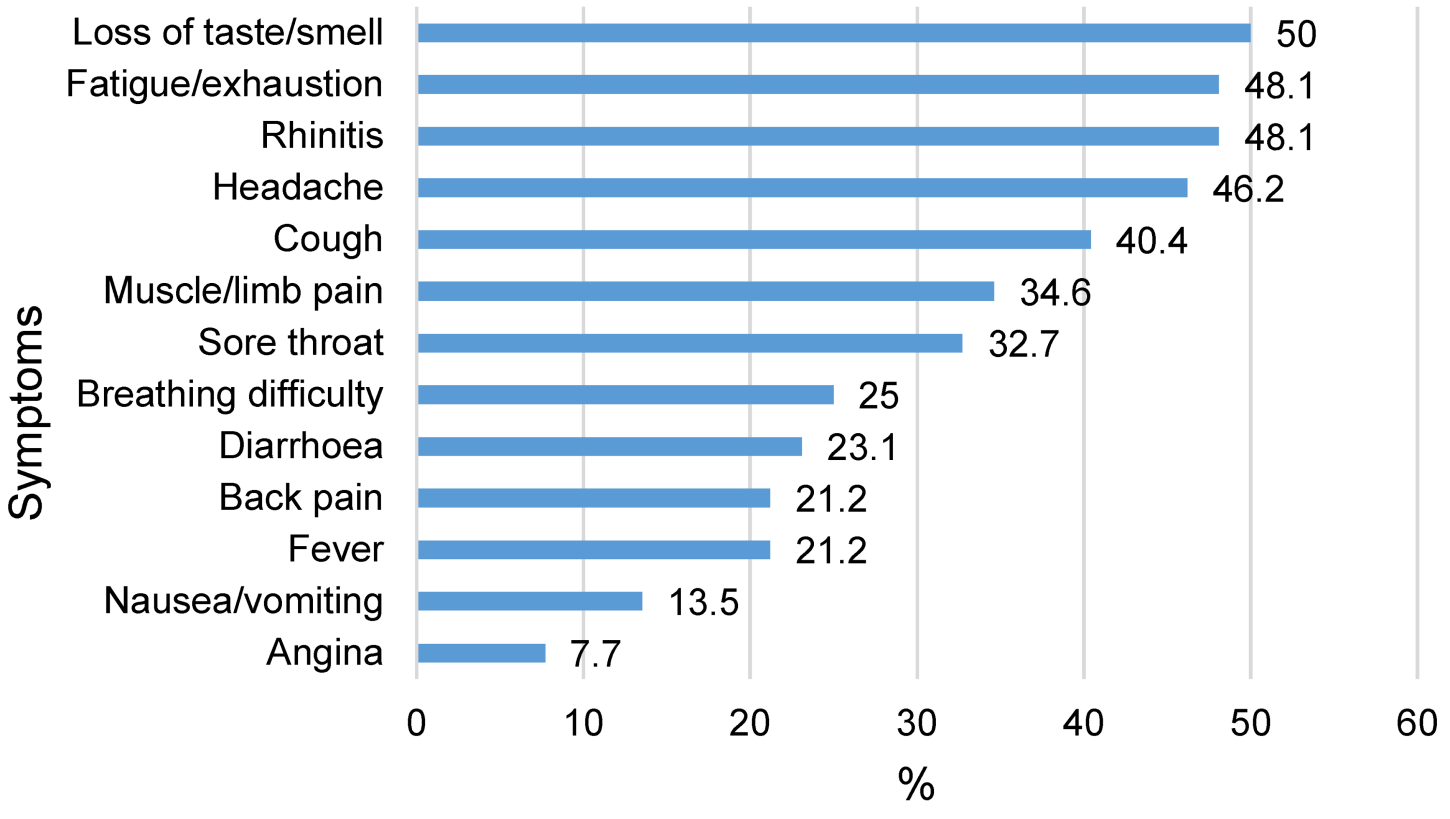
Symptoms of COVID-19 in PCR or IgG positive HW (n=52)

The most common symptoms among staff who tested positive were loss of taste and smell (50%), fatigue and exhaustion (48.1%), rhinitis (48.1%), headache (46.2%) and cough (40.4%) (Figure 1 (Fig. 1)). At the time of study, 11.5% of those who tested positive reported that they had not yet fully recovered, and 36.5% reported to have recovered. 52% gave no answer. The most common remaining symptoms were taste and smell disturbances (7.7%), exhaustion (5.8%), shortness of breath (5.8%) and dizziness (5.8%) (not shown in table). 

When comparing SARS-CoV-2 positive participants, depending on the IgG titre level, the most common symptoms with a titre of ≥0.8 to <1.5 were cough (43.8%), rhinitis (37.5%), headache (37.5%) and loss of taste/smell. Among participants with an IgG ≥1.5, the most common symptoms were loss of taste/smell (57.6%), fatigue/exhaustion (57.6%), rhinitis (51.5%) and headache (51.5%). Only the difference regarding fatigue and exhaustion (25.0% versus 57.6%) was statistically significant with respect to the IgG titre (Table 4 (Tab. 4)).

## Discussion

By means of antibody testing, this study investigated the development of SARS-CoV-2 infection during the first wave in Germany among the staff of a hospital. Out of 903 participants, 52 (5.8%) were infected. Despite being a low rate, it is higher than in comparable studies in Germany during the first wave. For example, 3.9% of healthcare staff were infected in Eschweiler (study period 27 April 2020–20 May 2020) [10]. In a study from Bonn (study period 9 March–30 April 2020), 1% of staff were infected [11]. The infection rate was 1.8% in Hamburg (study period 20 March–17 July 2020) and 1.6% in Essen (study period 25 March–21 April 2020), although in Essen, only staff who had direct contact with SARS-CoV-2 patients were tested [12], [13]. When compared across Europe, a study from Italy (Rome), where the virus first appeared in Europe (18 March–27 April 2020), indicated that 2.7% of the staff were infected [14]. The high infection rate of the Rhine-Maas Hospital is probably associated with its proximity to the Heinsberg district, which was severely affected by SARS-CoV-2 at the beginning of the first wave.

In terms of the frequency of the symptoms, there are parallels with other surveys: in the Bonn study, 72% of those infected had symptoms (50% in this study) [11]. The Eschweiler study found that 30.8% of those infected had symptoms [10], the most common being headache (30.3%), fatigue/exhaustion (30.3%), sore throat (28.8%) and cough (28.8%). Loss of taste/smell was only found in 3% of the cases. In the Hamburg study, the symptoms included rhinorrhoea (72.7%), headache (68.2%), muscle pain (59.1%) and cough (50%) [12]. It is worth noting that in our study 11% continued to have symptoms for more than four weeks, indicating Long COVID. 

The significantly increased risk among those returning from risk areas is also found in the Bonn study: 18% of the infected staff (n=56) had been to a risk area [11]. In the Eschweiler study, 60.5% of those infected had had contact with a positive case, and 36.8% for more than 15 minutes, with 28.9% wearing a facemask at work [10]. In the present study, 78.8% had contact, and 59.6% had contact for more than 15 minutes, with 67.3% of those infected reporting that they had worn a facemask at work. With regard to the effectiveness of protective measures, it should be taken into account that those infected with SARS-CoV-2 had a higher proportion of longer contact duration (31 of 52 [59.6%] versus 376 of 903 [41.6%]). 

Strikingly, in this study, the percentage of infected individuals is the highest among nursing assistants compared to the other occupational groups with patient contact. A cross-sectional study from China came to a similar conclusion and suggested that increased medical education plays a role in the prevention of the disease [15]. 

### Strengths and limitations of the study

The period of the study from 19.6.2020 to 17.7.2020 covers the first “Corona wave”, as the antibody tests conducted reflect the entire course of infection during this period. The location of the hospital near a risk area allows a good assessment of the risk of disease for such a case. Because of the high participation rate, this can be considered representative of the course of infection among hospital staff. 

Before the study started, 23 participants already knew about their disease, so there is a risk of bias, for instance, due to selecting symptoms typical of SARS-CoV-2 when answering the questions. Due to the heightened risk, hospital volunteers were given time off from work shortly after the pandemic began. They only came to the hospital for testing. This would mean that the volunteers would have no occupational infection risk during the time up to testing, compared to the rest of the staff. Furthermore, due to the low infection rate, the number of infected HW was small. This did not permit extensive analysis of risk factors. Nevertheless, work-related risk factors were ob-served. This knowledge might be useful for assessing claims of COVID-19 as occupational disease [16]. 

### Outlook and personal comments by the occupational health doctor (A. E-A)

This study addressed the impact of the first “Corona wave” on the staff of a hospital. At the time, there was still some uncertainty about effective protective measures. For instance, the staff on the “Corona ward” frequently rotated; consequently, there was a risk of carryover to other areas of the hospital. It should also be noted that each “wave” is different: the “third wave” in particular, characterised by the British variant, has led to increased infections in the RMK (105 staff members from November to April) despite increased protective measures (personal communication). However, since the start of the vaccination campaign for healthcare staff, the number of tests has decreased as fewer staff members have shown symptoms. Since May 2021, only one staff member (who did not wish to be vaccinated) tested positive, which clearly speaks for the success of vaccination. Now, booster vaccinations are being administered at the hospital, as some vaccine breakthroughs have been observed.

### Conclusions and implications for practice

Although the RMK was serving patients from one of the first hot spots in Germany, the infection rate after the first wave of the pandemic was rather low. Nevertheless, HW with contact to patients with COVID-19 had an increased infection risk after controlling for private risk factors. This should be considered regarding the assessment of COVID-19 as an occupational disease. Most of the HW reported contact to COVID-19 patients in our study. An improved emergency plan might help reduce contacts to a limited number of HW. PPE did not show a protective effect in this study. Improving availability of PPE and exercising the correct use of PPE might be helpful in the future to better protect HW from infection. 

## Notes

### Competing interests

The research work of CVcare at the University Medical Centre Hamburg-Eppendorf (UKE) is primarily funded by the BGW. The BGW had no part in the design of the study, the collection, analysis, or interpretation of data, writing the manuscript, or the decision to publish the results. The other authors from the RMK state that there are no conflicts of interest.

### Acknowledgement 

The study was funded by the Statutory Accident Insurance and Prevention in the Health and Welfare Services (BGW) in Hamburg.

We would like to thank the study team at the Rhine-Maas Hospital, especially the staff, for their participation in the study.

## Figures and Tables

**Table 1:**
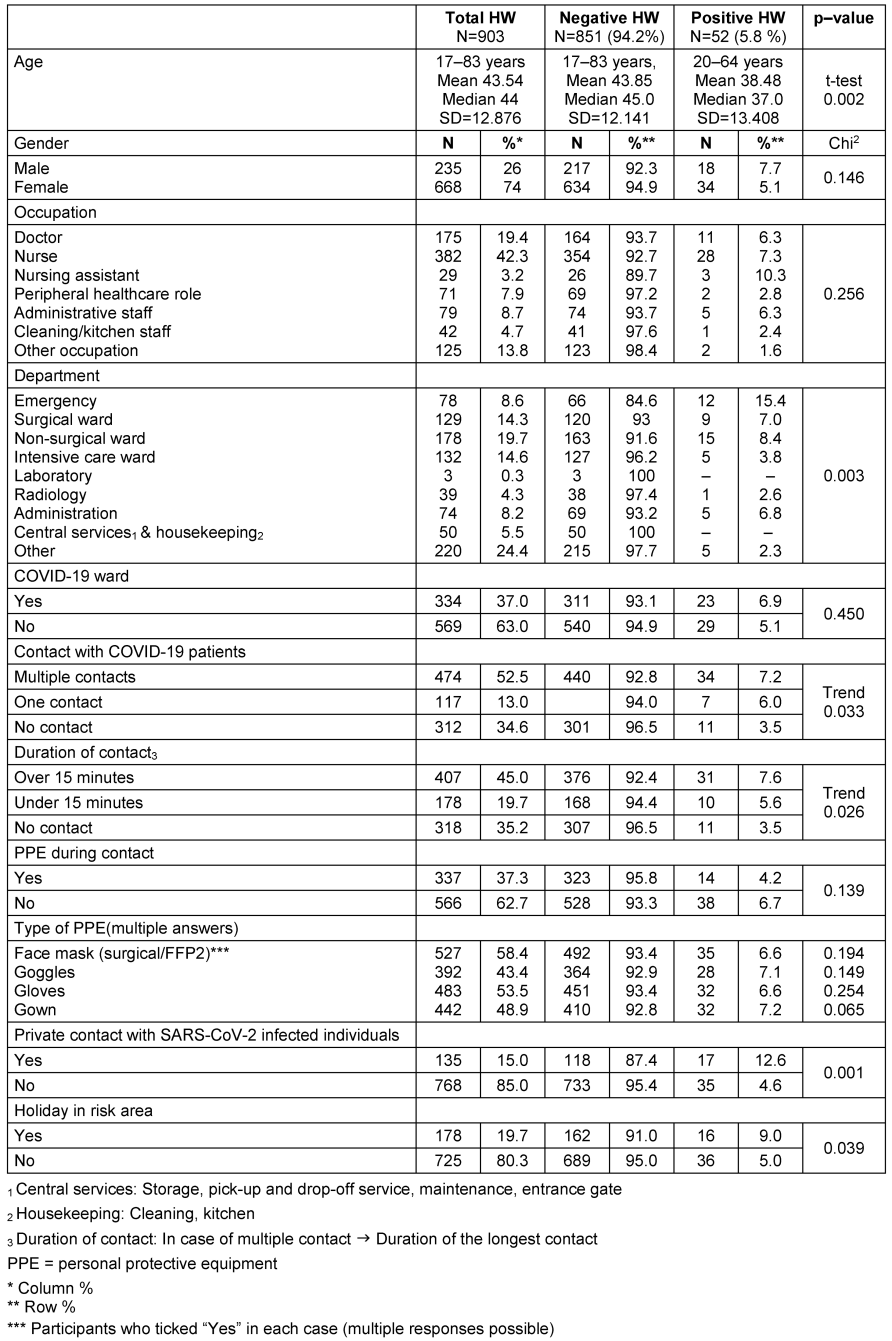
Description of the study population and comparison of negatively vs. positively tested HW

**Table 2:**
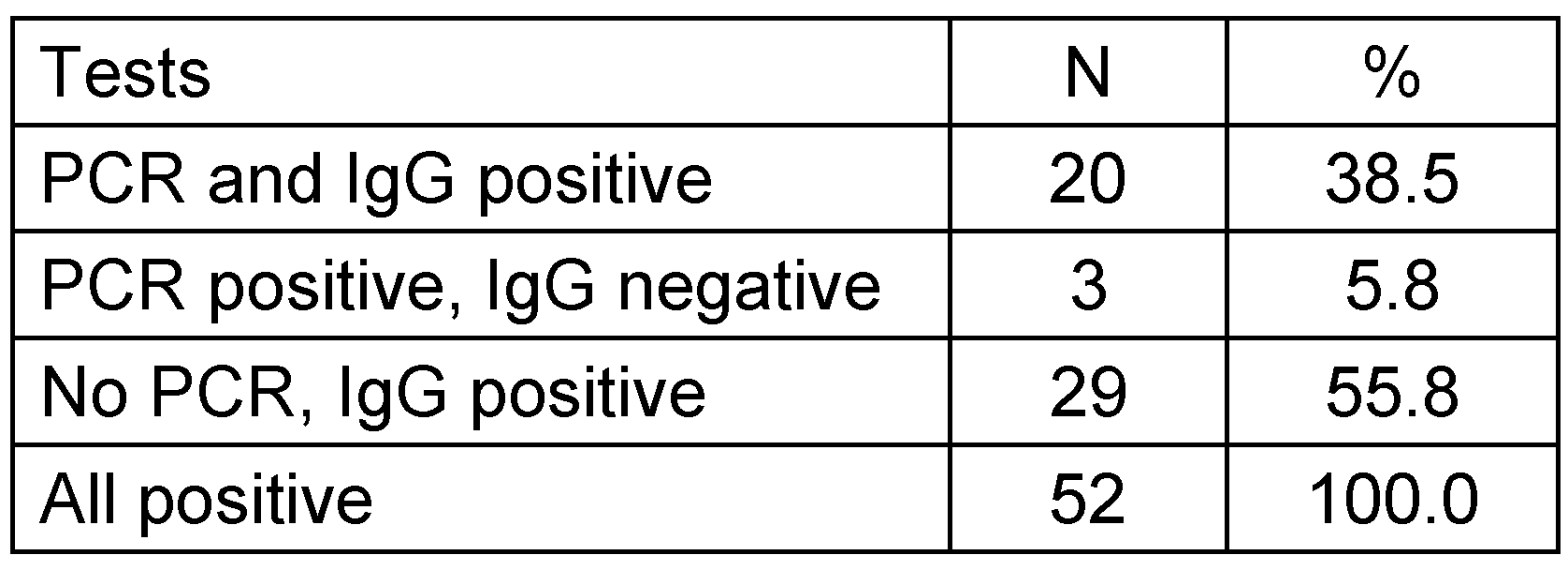
Staff with positive PCR or positive serology result

**Table 3:**
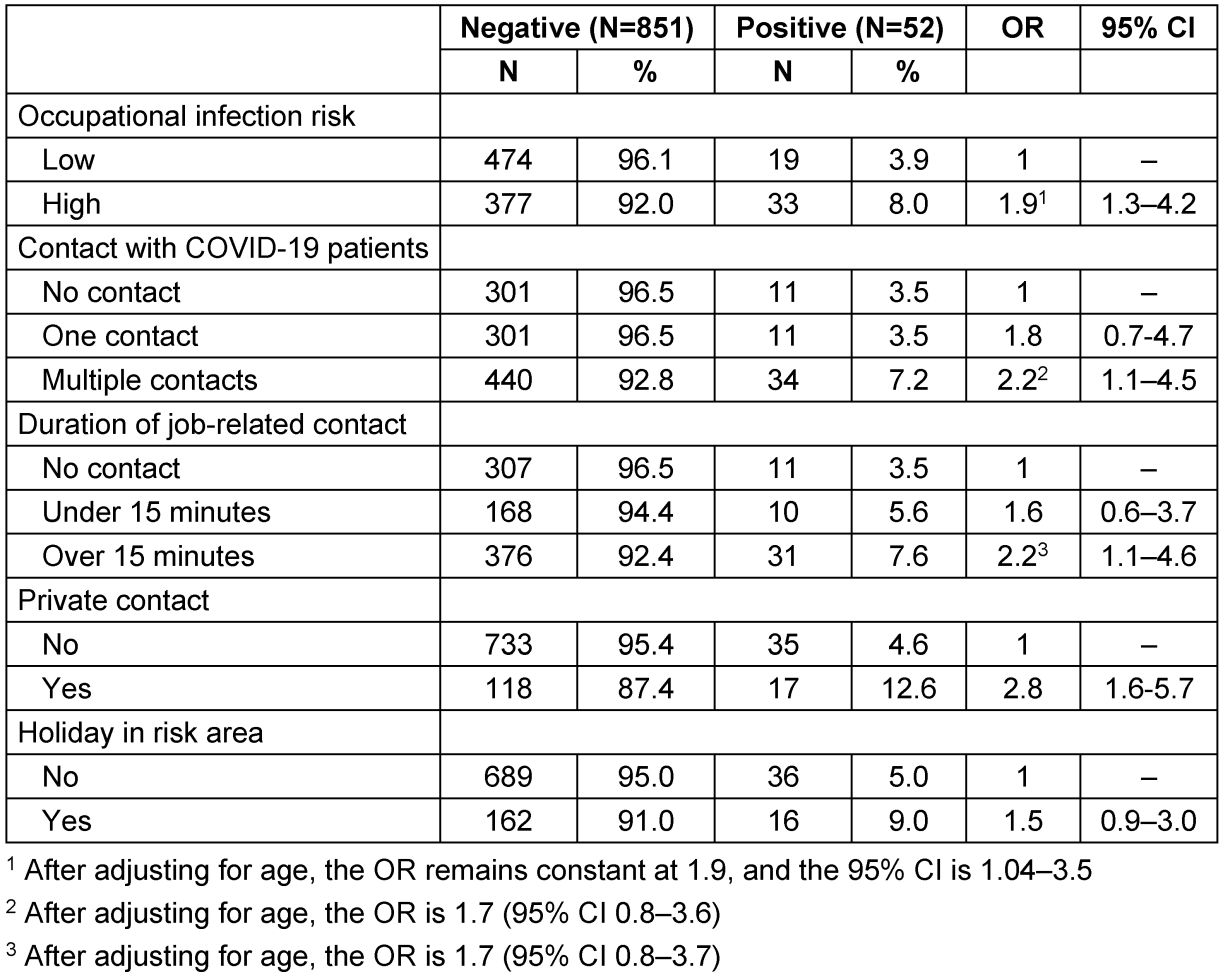
Risk factors for a SARS-CoV-2 infection

**Table 4:**
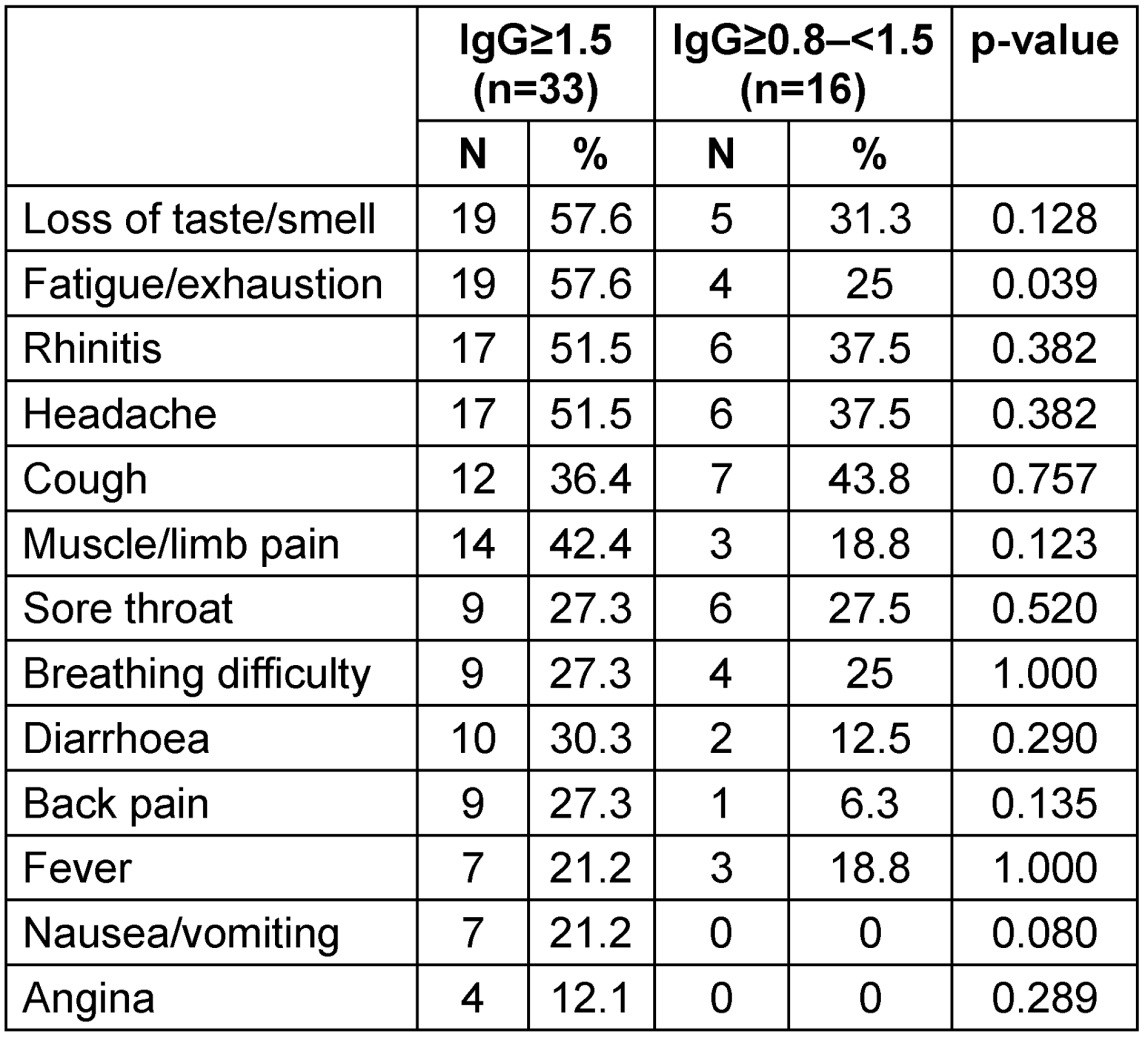
Symptoms depending on titre level
